# Recalcitrant lichen planus pigmentosus treated with topical ruxolitinib

**DOI:** 10.1016/j.jdcr.2023.10.012

**Published:** 2023-10-31

**Authors:** Hannah L. Cornman, Elena Wei, Jaya Manjunath, Emily Z. Ma, Brenda Umenita Imo, Alexander L. Kollhoff, Anusha Kambala, Jingyi Zhang, Shivani S. Patel, Shawn G. Kwatra

**Affiliations:** a Department of Dermatology, Johns Hopkins University School of Medicine, Baltimore, Maryland; b Anne Arundel Dermatology, Towson, Maryland; c Department of Oncology, Johns Hopkins University School of Medicine, Baltimore, Maryland

**Keywords:** cream, hyperpigmentation, JAK inhibitor, janus kinase inhibitor, lichen planus pigmentosus, recalcitrant, ruxolitinib, topical

## Introduction

Lichen planus pigmentosum (LPP) is a rare, pigmented variant of lichen planus that is characterized by hyperpigmented, dark brown to gray-black macules in sun-exposed areas of the body such as the face, neck, trunk, and flexures.^1^ This condition can cause considerable distress and a reduction in quality of life, but there are currently no approved therapies for LPP. Furthermore, existing treatments such as topical and oral steroids, topical calcineurin inhibitors, and topical retinoids, have limited success.^2^

LPP is an interface dermatitis thought to be caused by a type IV hypersensitivity reaction to an unknown antigen with lichenoid inflammation, leading to keratinocyte destruction, melanin incontinence, and superficial dermal pigmentation.^1^ This inflammation and keratinocyte susceptibility to destruction is largely driven by interferon-γ signaling through the Janus kinase (JAK) -signal transducer and activator of transcription pathway, with interferon-γ-induced chemokines (CXCL9, CXCL10, and CXC11) shown to play a major role in attracting the inflammatory cell infiltrate in interface dermatitis.^3^^,^^4^ Therefore, JAK inhibitors such as ruxolitinib, which have known inhibitory effects on CXCL9/10/11-CXCR3 and STAT signaling pathways, represent promising therapeutic candidates for treatment of LPP.^5^

In this case study, we present the unique case of a patient with recalcitrant LPP who exhibited a substantial improvement after treatment with topical ruxolitinib 1.5% cream. This suggests the potential effectiveness of ruxolitinib as a treatment option for LPP, offering hope for improved management of this challenging condition.

## Case report

**Fig 1:**
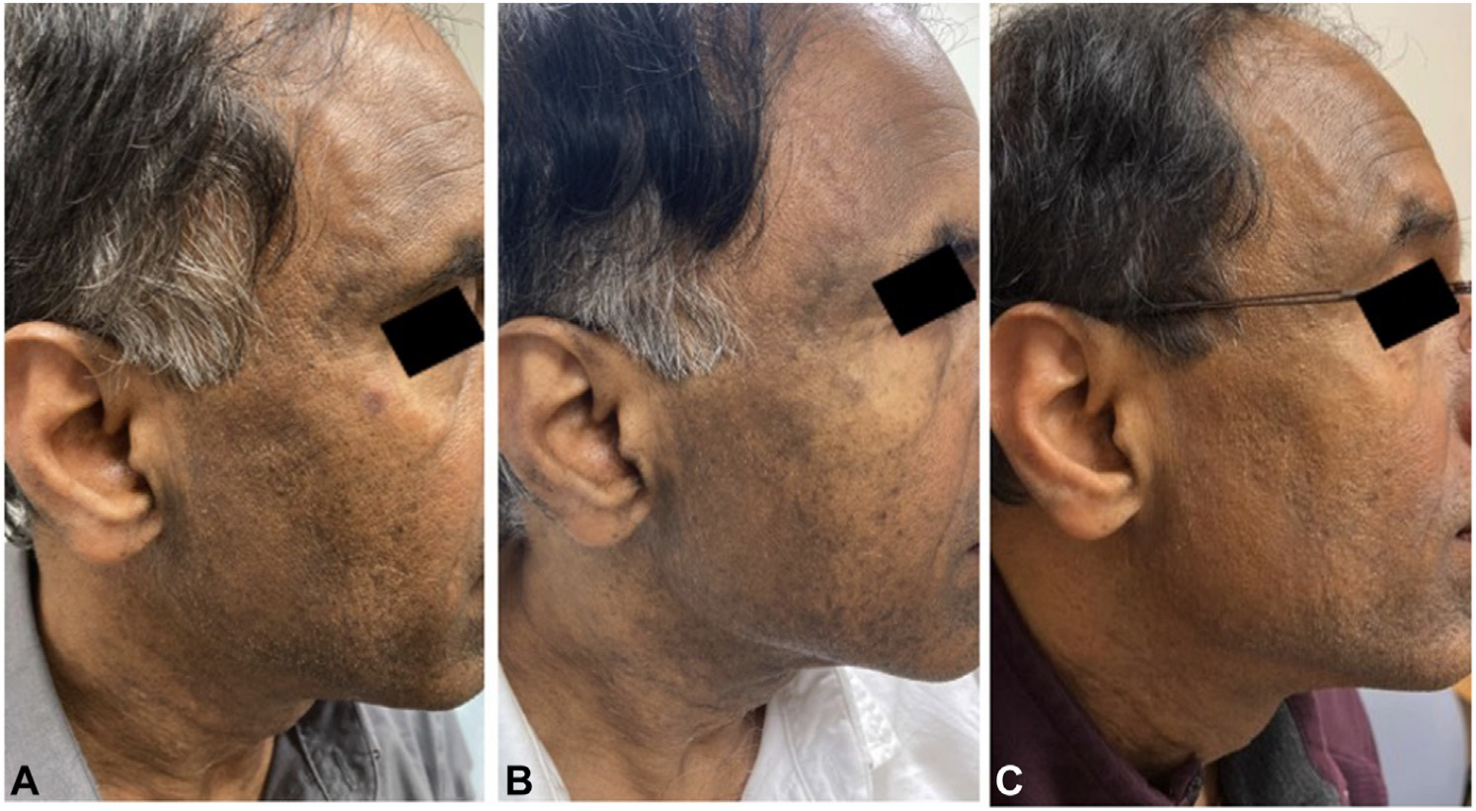
Clinical improvement of lichen planus pigmentosus skin lesions during treatment with topical ruxolitinib 1.5% cream. **A,** Clinical photographs of skin lesions at initial presentation with gray-violaceous hyperpigmented macules coalescing into patches on the bilateral cheeks extending to the temples and anterior neck. **B,** Significantly reduced hyperpigmentation and smaller affected area after 3 months of treatment. **C,** Further reduction of hyperpigmentation after 9 months of treatment.

A 63-year-old male presented for evaluation of a rash on the bilateral cheeks since at least 5 years prior, associated with significant pruritus (worst itch numeric rating scale [WI-NRS] score 8/10) and loss of facial hair. The rash and itch were recalcitrant to topical steroids, calcineurin inhibitors, ketoconazole cream, hydroquinone, and 3 months of oral minocycline. Physical examination found multiple gray-violaceous hyperpigmented macules coalescing into patches on the bilateral cheeks extending to the temples and anterior neck (Fig 1, *A*). Skin biopsy of the left submandibular area revealed interface dermatitis with pigment incontinence, consistent with LPP. The patient was prescribed ruxolitinib 1.5% cream to be applied topically twice daily, with continued diligent photoprotection. Three months later, the patient reported significant improvement, including stabilization of hyperpigmentation and mild lightening of the affected area (Fig 1, *B*). Nine months after initiating topical ruxolitinib therapy, the patient reported a significant decrease in hyperpigmentation and pruritus (WI-NRS score 2/10) (Fig 1, *C*). The patient denied experiencing any side effects, including skin tolerability issues (eg stinging/burning sensation).

## Discussion

This case suggests that topical ruxolitinib can be a low-risk, effective therapy for LPP, which is in line with growing evidence of dysregulated JAK-signal transducer and activator of transcription signaling in this condition.^1^^,^^3^^,^^4^ This is clinically significant because LPP is notoriously difficult to treat, and there is limited data on effective therapeutic options. Small prospective analyses have investigated the efficacy of topical tacrolimus,^2^ oral isotretinoin, oral tranexamic acid, and Q-switched Nd:YAG lasers in the treatment of LPP with some success.^6^ However, these studies have small sample sizes, and most had a significant number of patients who did not improve, demonstrating the need for further investigation of novel LPP therapies.

Topical ruxolitinib presents a compelling addition to the dermatologist’s armamentarium for the treatment of LPP for several reasons. Already approved by the United States Food and Drug Administration for nonsegmental vitiligo and atopic dermatitis,^7^ this medication stands out for its safety on delicate skin areas like the face and neck, which are some of the most frequently affected areas in LPP.^1^^,^^8^ In contrast, other commonly used topical treatment options for LPP such as topical steroids or calcineurin inhibitors pose challenges for facial application due to the risk of skin atrophy, acne, and burning sensations. Additionally, topical ruxolitinib also led to significant improvement in the patient’s pruritus, which is consistent with prior studies in atopic dermatitis patients.^8^^,^^9^

Future prospective studies and clinical trials are needed to investigate the safety and clinical efficacy of topical ruxolitinib 1.5% cream in treating recalcitrant LPP as well as to determine the optimal dosing and duration of treatment. In conclusion, this case sheds light on the promising role of topical ruxolitinib in the management of lichen planus pigmentosus, emphasizing its potential to be explored as a novel therapeutic option in patients with recalcitrant disease.

## Conflicts of interest

Dr Kwatra is an advisory board member/consultant for Abbvie, Aslan Pharmaceuticals, Arcutis Biotherapeutics, Castle Biosciences, Celldex Therapeutics, Galderma, Genzada Pharmaceuticals, Incyte Corporation, Johnson & Johnson, Leo Pharma, Novartis Pharmaceuticals Corporation, Pfizer, Regeneron Pharmaceuticals, and Sanofi and has served as an investigator for Galderma, Incyte, Pfizer, and Sanofi. All others declare no conflict of interest to declare. Dr Patel is an advisory board member/consultant for Arcutis Biotherapeutics, Dermavant, Incyte, Sanofi, and Regeneron.
